# Rational design of supramolecular hemin/G-quadruplex–dopamine aptamer nucleoapzyme systems with superior catalytic performance†
†Electronic supplementary information (ESI) available: Oxidation of ABTS^2–^, additional figures of computational simulations, kinetic curves, and parameters of the switchable system. See DOI: 10.1039/c5sc04832j


**DOI:** 10.1039/c5sc04832j

**Published:** 2016-01-25

**Authors:** H. Bauke Albada, Eyal Golub, Itamar Willner

**Affiliations:** a Institute of Chemistry , The Minerva Center for Biohybrid Complex Systems , The Hebrew University of Jerusalem , Jerusalem , 91904 , Israel . Email: willnea@vms.huji.ac.il ; Fax: +972-2-6527715 ; Tel: +972-2-6585272

## Abstract

The rational design of a set of hemin/G-quadruplex (hGQ)–dopamine binding aptamer (DBA) conjugates, acting as nucleoapzymes, is described. The nucleoapzyme constructs consist of a hGQ DNAzyme as a catalytic unit and DBA as a substrate binding unit that are brought into spatial proximity by a duplex scaffold composed of complementary oligonucleotide strands. When the hGQ unit is linked to the duplex scaffold *via* a single-strand DNA tether of variable length, the resulting nucleoapzymes reveal a moderate catalytic enhancement toward the H_2_O_2_-mediated oxidation of dopamine to aminochrome as compared to the process stimulated by the separated hGQ and DBA units (5–7 fold enhancement). This limited enhancement is attributed to inappropriate spatial positioning of the hGQ in respect to the dopamine binding site, and/or to the flexibility of the tether that links the hGQ catalytic site to the double-stranded scaffold. To solve this, rigidification of the hGQ/DBA conjugates by triplex oligonucleotide structures that anchor the hGQ to a duplex domain associated with the DBA units was achieved. By the sequential, programmed, triplex-controlled rigidification of the hGQ/DBA structure, a nucleoapzyme with superior catalytic activity toward the oxidation of dopamine to aminochrome is identified (30-fold catalytic enhancement). Molecular dynamics simulations reveal that in the resulting highly active rigidified nucleoapzyme structure, the hGQ catalytic site is positioned in spatial proximity to the opening of the DBA substrate binding site, thus rationalizing and supporting the enhanced catalytic functions of the system. Finally, the most active nucleoapzyme system was subjected to fuel- and anti-fuel strands that separate and re-assemble the nucleoapzyme structure, allowing “ON” and “OFF” switching of the nucleoapzyme catalytic functions.

## Abstract 2

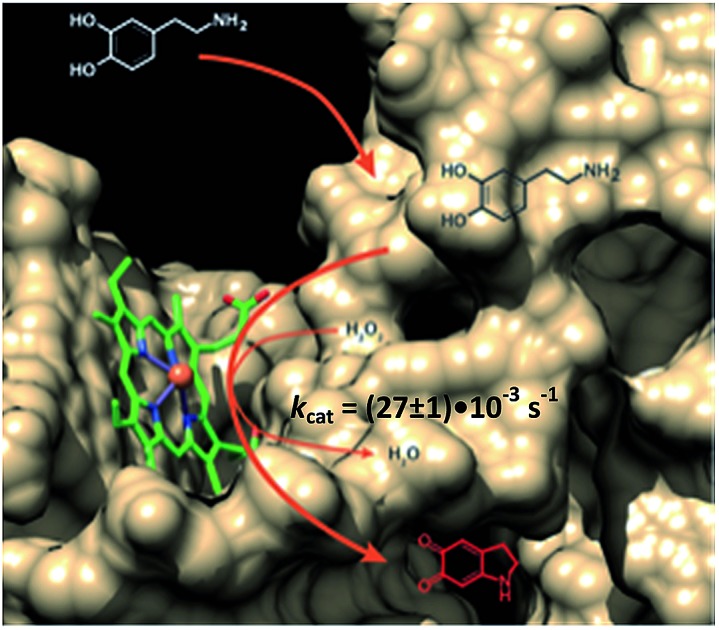
We designed supramolecular nucleoapzyme systems displaying enhanced dopamine-oxidizing abilities using computational simulations, and developed a system having switchable catalytic activities.

## Introduction

Catalytic nucleic acids (DNAzymes) attract much research interest as a new class of catalytic biomaterials.^1^ Numerous chemical transformations are catalyzed by DNAzymes,^2^ which include nicking and ligation of oligonucleotide substrates,^3^ phosphorylation of hydroxyl-substituted substrates,^4^ the C–C bond forming Michael addition reaction^5^ and Diels–Alder reaction,^6^ and more.^7^ One of the most studied DNAzymes is the hemin/G-quadruplex (hGQ) horseradish peroxidase mimicking DNAzyme.^8^ Similar to the native enzyme, this DNAzyme catalyzes the H_2_O_2_-mediated oxidation of organic substrates to chromophoric^9^ or fluorescent^10^ products, or catalyzes the generation of chemiluminescence in the presence of luminol and H_2_O_2_.^11^ Also, this DNAzyme catalyzes biologically relevant reactions such as the H_2_O_2_-mediated oxidation of phenols,^12^ thiols,^13^ NADH,^14^ and aniline,^15^ and the aerobic oxidation of thiols or NADH.^14^ Apart from these transformations, much research has been directed for the application of DNA as a chiral scaffold for homogeneous catalysts, not only in the previously mentioned C–C bond forming reactions, but also in allylation, hydrogenation, and hydroformylation reactions.7a,^16^ Clearly, DNA is a valuable material for many catalytic transformations.

The catalytic functions of DNAzymes were widely applied for the development of amplified electrochemical and optical sensors,^17^ for the construction of DNA computing systems and logic gate circuits,^18^ for the synthesis of stimuli-responsive nanoparticle carriers for controlled drug release^19^ and programmed synthesis,^20^ for the triggered dissolution of hydrogels^21^ and the activation of enzymatic cascades,^22^ and for the assembly of optoelectronic systems^23^ and switchable DNA machines.^24^ In spite of the significant progress in the development of DNAzymes and their broad applications, DNAzymes that operate on non-nucleotide substrates often suffer from lower activities as compared to native enzymes. This presumably, originates from the fact that such DNAzymes lack binding sites for their substrates, and thus do not possess means to concentrate substrates at the active site. Even more, appropriate alignment of the catalytic unit with respect to the binding site for the substrate could possibly hamper efficient catalysis. In this paper, we describe how such alignment is realized. A possible method to improve the catalytic functions of DNAzymes, and eventually to develop a new approach to design catalytic nucleic acids, rests on the application of sequence-specific ligand-binding oligonucleotides (aptamers). Aptamers are selected by the SELEX process,^25^ and display specific binding affinities toward foreign molecules.^26^ The selective binding properties of aptamers have been widely utilized for the development of sensors and for biomedical applications.^27^ In a previous study, we argued that the conjugation of aptamers to DNAzymes could yield hybrid structures that combine the active site and recognition site into an integrated structure, resulting in enhanced catalytic functions.^28^ In fact, one may envisage the extension of this concept by the binding of synthetic catalysts (*e.g.* metal complexes or catalytic ligands) to aptamers to yield new catalytic nucleic acids.

**Fig. 1:**
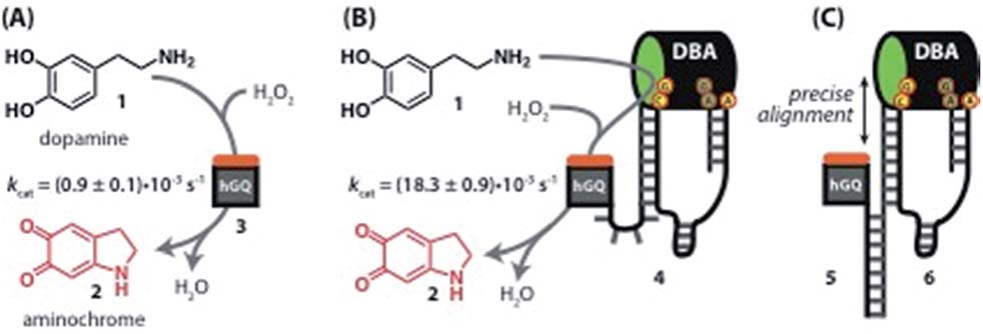
(A) Oxidation of dopamine (**1**) to aminochrome (**2**) catalyzed by the hemin/G-quadruplex (hGQ) DNAzyme (**3**) and H_2_O_2_. (B) 20-fold enhanced oxidation of dopamine (**1**) to aminochrome (**2**) by a nucleoapzyme that contains the hGQ DNAzyme unit and the dopamine binding aptamer (DBA) unit integrated in one oligonucleotide strand (**4**). (C) Alignment of the catalytic unit (hGQ, **5**) and dopamine binding unit (DBA, **6**) by supramolecular double-strand association.

Recently, we reported the H_2_O_2_-mediated hemin/G-quadruplex (hGQ) DNAzyme-catalyzed oxidation of dopamine (**1**) to aminochrome (**2**) (Fig. 1A).^28^ Furthermore, we introduced a series of hemin/G-quadruplex–dopamine binding aptamer conjugates hGQ–DBA hybrid structures, termed by us “nucleoapzymes”, as catalytic conjugates for the enhanced catalytic oxidation of dopamine (**1**) to aminochrome (**2**) (Fig. 1B). Indeed, we demonstrated that concentrating the substrate at the hGQ active site by means of the aptamer function leads to significantly higher rates for this oxidation reaction when compared to the reaction in the presence of the separated DNAzyme and DBA units. We also demonstrated that the modes of linking the DBA to the hGQ, and the introduction of base mutations into the nucleoapzymes strongly affect the catalytic features of the nucleoapzymes.^28^ Clearly, covalent conjugation of DNAzyme/aptamer units represents a method to integrate binding and catalytic sites into a hybrid structure exhibiting improved catalytic functions. One may envisage, however, tunable means to align the aptamer binding site in respect to the active site, *e.g.* with a duplex-assisted assembly of an aptamer–hGQ supramolecular nucleoapzyme structure (Fig. 1C). According to this approach, both the DNAzyme and the aptamer binding site are extended by nucleic acid tethers that exhibit base complementarities. Formation of the duplex between the respective tethers leads to tunable spatial proximities between the binding and catalytic sites. Additionally, Hoogsteen-type triplex interactions between the hGQ strand and the aptamer strand can be designed, leading to rigidification of the hGQ/aptamer supramolecular structure, and thus to improved catalytic activities. Here we wish to report this rational design of supramolecular hGQ/dopamine nucleoapzyme structures, and the assembly of hGQ/aptamer assemblies of controllable superior activities. Experimental results are rationalized by molecular dynamic simulations; a system with switchable catalytic performance is also presented.

## Results and discussion

### Rational design of the supramolecular nucleoapzyme systems

**Fig. 2:**
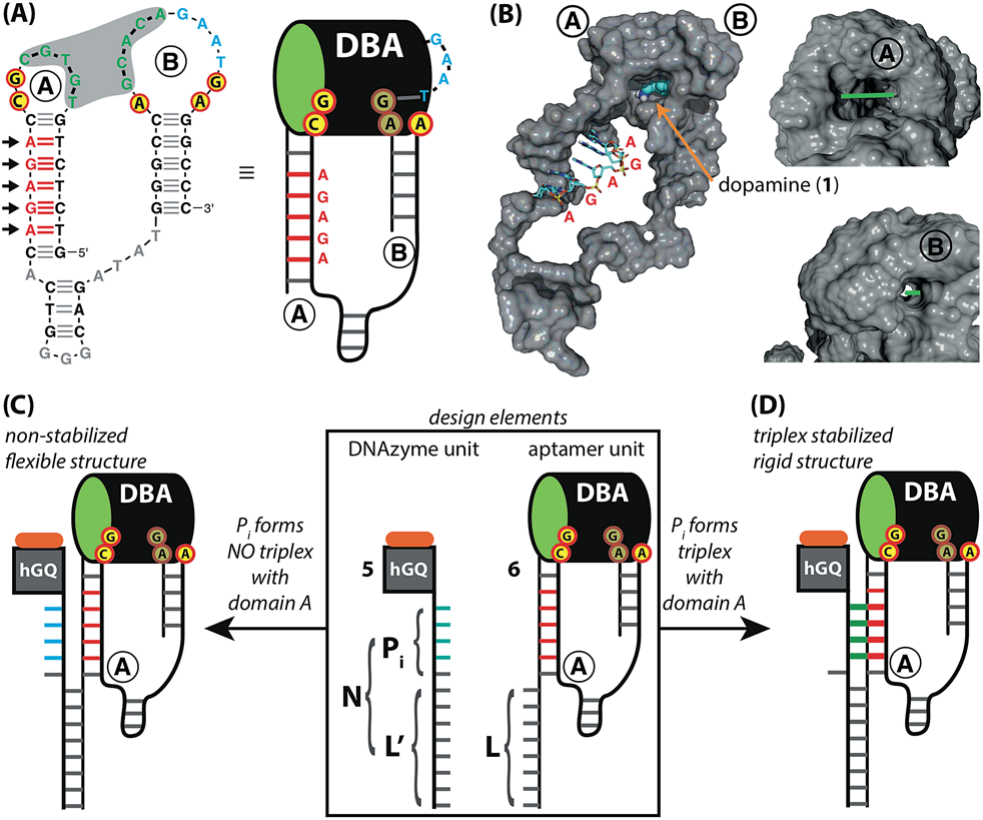
(A) Primary structure of the dopamine binding aptamer (DBA). The two largest hairpin structures, A and B, form the dopamine binding site upon hybridization of the five consecutive complementary bases in the loops (shown in green, highlighted by the grey background). The red-and-yellow circles highlight the residues that form the binding site for dopamine; the GAAT-sequence that blocks the 3′-end facing side of the DBA is shown in blue. Also shown are the five consecutive bases in hairpin A that are available for triplex formation (in red); the arrows indicate the direction from which the triplex-forming strand binds. On the right side, the schematic depiction of the DBA that is used in this study is shown. (B) Computer-generated model of the entire DBA structure. Hairpins A and B are indicated, as well as a docked dopamine ligand (shown by the arrow); the five bases that are available for triplex DNA formation are shown without their van der Waals surface. The asymmetric nature of the tunnel-shaped binding pocket is shown on the right, with the 5′-end facing wide opening (top, bar length = 8 Å) and narrow 3′-end facing opening (bottom, bar length = 2 Å). Access of the binding site for the dopamine substrate is from the wide 5′-end facing opening. (C) and (D) Design elements of the supramolecular DNA structures used in this study (highlighted by the rectangle. The GAAT-sequence is left out for clarity). The extensions on the hGQ DNAzyme (**5**) and DBA (**6**) units, L′ and L, respectively, are complementary to each other. Positioning the hGQ catalytic unit with respect to the wide opening of the dopamine binding site is tuned by a programmable unit P_*i*_ that is inserted in between L′ and the hGQ catalytic unit. When P_*i*_ does not form a triplex unit with the stem of hairpin A, a flexible tether connects the L/L′ duplex and the catalytic unit (C). However, as shown in (D), when P_*i*_ does form a triplex unit with the stem of hairpin A, the catalytic unit is immobilized on the stem of hairpin A.

The design for the supramolecular hGQ/aptamer hybrids for the oxidation of dopamine (**1**) to aminochrome (**2**) is illustrated in Fig. 2. Analysis of the intra-chain hybridization of the DBA sequence indicates that it forms two main hairpin domains, A and B, where interactions between the kissing loops of the two hairpins yield the dopamine binding pocket (Fig. 2A).^29^ Base-mutations studies showed that the nucleotides GC (associated with hairpin A) and AGA (associated with hairpin B) play a key-role in binding dopamine to the aptamer.^30^ Furthermore, the non-hybridized GAAT-sequence in the loop of hairpin B, depicted in blue in Fig. 2A, blocks the 3′-end-facing side of the binding pocket. Indeed, computational simulations of the dopamine binding aptamer reveal the presence of a wider opening on the side of hairpin A (Fig. 2B). Thus, only the opening of the binding pocket that faces the 5′-end of the aptamer allows the association of the dopamine substrate. Furthermore, it should be noted that five of the seven nucleotide bases in the stem of hairpin A are available to form triplex-hybridized DNA structures (the red letters in Fig. 2A and B). Accordingly, the 5′-end of the aptamer was extended with a tether L; the hemin/G-quadruplex structure was extended with a tether N that included two domains, L′ and P_*i*_ (Fig. 2C). Sequence L′ is fully complementary to L, both of which contain 18 nucleotides with a fixed composition, whereas domain P_*i*_ represents a single strand of programmable composition and length, denoted by *i*. Thus, hybridization between domains L/L′ yields the duplex structure that provides the supramolecular nucleoapzyme scaffold, where the hGQ active site is in spatial proximity to the dopamine binding site. Additionally, domain P_*i*_ is of programmed length and thus allows tunable spatial positioning of the hGQ DNAzyme relative to the binding site. The single strand tether P_*i*_ is, however, flexible and a firm spatial positioning of the hGQ DNAzyme in respect to the binding site, a favored configuration for enhanced catalysis, is not controlled. In order to overcome this limitation, we made use of the fact that programmable sequences in the DNA may provide a code to generate secondary structures capable of bringing together the hGQ catalytic site and the dopamine binding site. Specifically, we introduce into the P_*i*_ domain a base code to form triplex structures between P_*i*_ and the stem of hairpin A of the DBA (Fig. 2D). Such triplex assisted juxtaposition of the catalytic and binding sites of nucleoapzymes could provide means to closely mimic enzyme functions as it allows precise positioning of the two active units that are essential for catalysis.

**Fig. 3:**
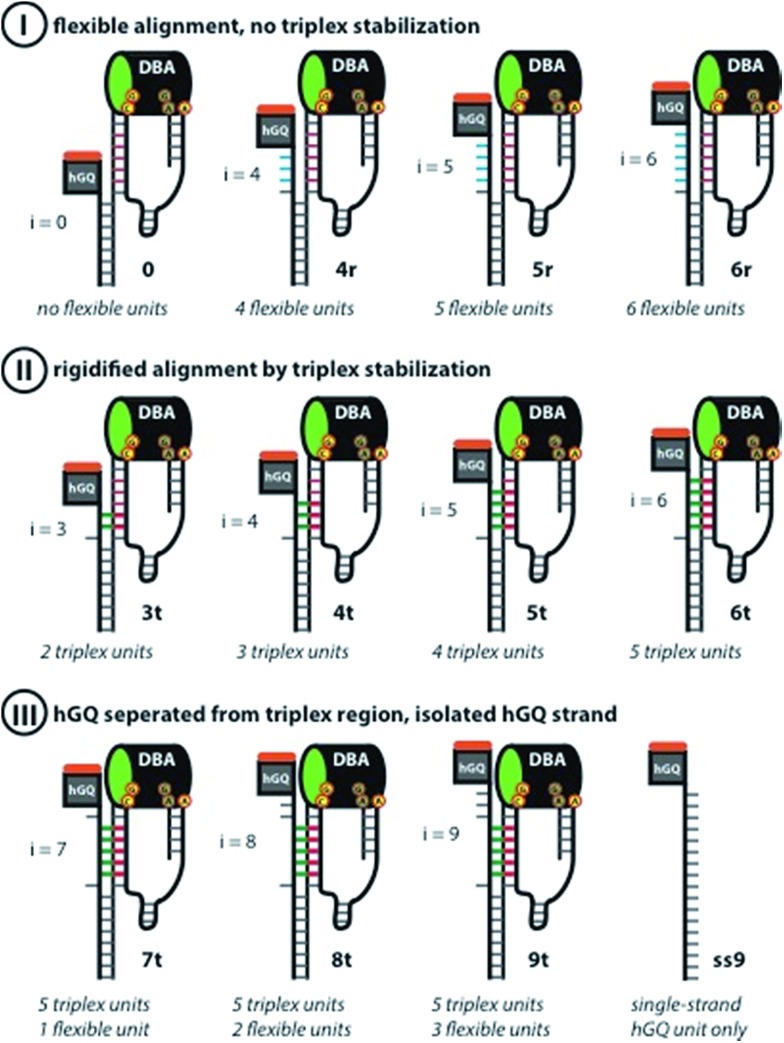
Schematic display of the set of rationally designed supramolecular hGQ DNAzyme dopamine binding aptamer (DBA) nucleoapzyme systems that were implemented in this study. Panel I: schematic depiction of the supramolecular nucleoapzyme structures that contain no P_*i*_ unit (system **0**), or flexible units between the double-strand L/L′ region and the hGQ and DBA units (systems **4r**, **5r**, and **6r**). The inability of the hGQ-strands to form a triplex with the stem of hairpin A is shown by the blue bars. Panel II: schematic depiction of nucleoapzyme structures in which the hGQ strand is fixed on the stem-region of hairpin A (**3t**, **4t**, **5t**, and **6t**). In each structure, the green bars show the DNA-triplex section between the hGQ strand and the stem of hairpin A. Panel III: control systems that were used in this study, *i.e.* supramolecular architectures that contain bases in addition to the triplex-section (**7t**, **8t**, and **9t**), and the single-strand extended hGQ structure **ss9**. In all DBA structures, the nucleotides that form the binding site are indicated; the green oval in the DBA highlights the 5′-end facing opening of the binding pocket.

According to these considerations the set of nucleoapzyme structures, shown schematically in Fig. 3, were constructed, and the catalytic activities of the structures were evaluated. Fig. 3, panel I, shows the schematic configurations of three hGQ/aptamer nucleoapzyme structures that either exclude P_*i*_ (*i* = 0), or include a flexible P_*i*_ single-stranded chain of variable length (*i* = 4, 5, and 6) that links the hGQ to the duplex L/L′, but that does not form a triplex structure with the stem-region of hairpin A. Fig. 3, panel II, depicts the schematic configurations of nucleoapzyme hGQ/aptamer structure rigidified by 2–5 triplex units between the P_*i*_ domain of hGQ carrying strand (with *i* = 3–6) and the stem of hairpin A. Fig. 3, panel III includes nucleoapzyme structure where domain P_*i*_ in the strand carrying the hGQ includes always five rigidifying triplex units yet the hGQ catalytic sites are separated from this triplex-domain by additional 1–3 nucleotide bases. The structure of the single-stranded hemin/G-quadruplex DNAzyme unit (**ss9**) is also shown. The catalytic activities within this set of nucleoapzymes will be discussed.

### Nucleoapzyme-catalyzed oxidation of dopamine to aminochrome

**Fig. 4:**
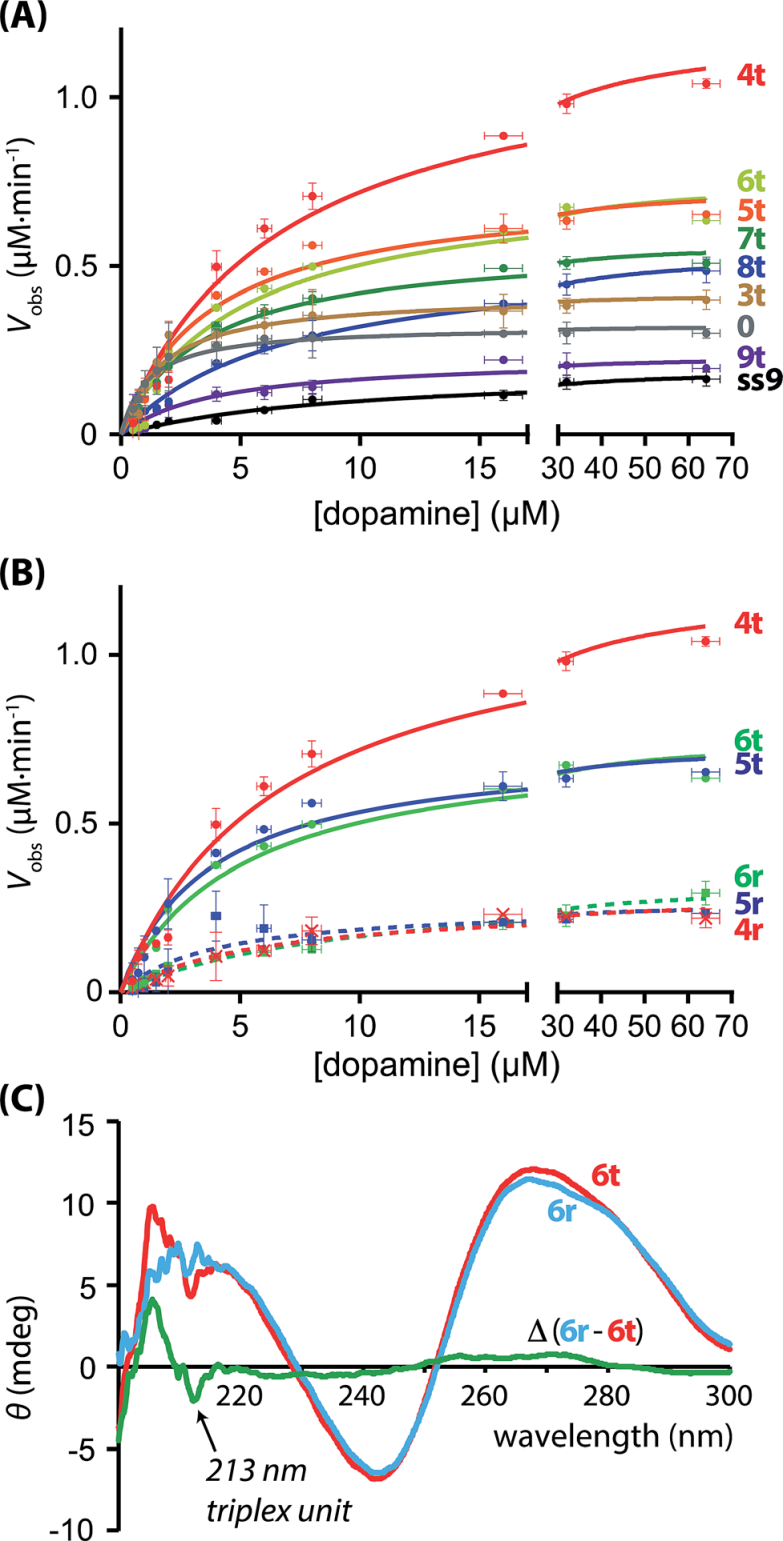
(A) Kinetics curves of the supramolecular nucleoapzyme systems that contain the triplex-DNA structure (**4t–9t**), including the curve of the structure with P_*i*_ = 0 (**0**), and that of the hGQ DNAzyme unit only (**ss9**). (B) Kinetics curves of the supramolecular nucleoapzyme systems that contain the triplex-DNA structure (**4t**, **5t**, and **6t**), and those that do not contain this stabilizing entity (**4r**, **5r**, and **6r**). (C) CD spectra of triplex-containing structure **6t** (red curve) and random-species **6r** (blue curve); the difference Δ between the two spectra reveals the presence of the triplex DNA feature at 213 nm (green line).

The hGQ-catalyzed oxidation of dopamine (**1**) to aminochrome (**2**) was stimulated in the presence of H_2_O_2_. All supramolecular hGQ DNAzyme-dopamine binding aptamer (DBA) structures that are subject of this study catalyze this conversion more efficiently than the single-strand hGQ unit (**ss9**) alone (entry 1–11 *versus* entry 12, Table 1). Fig. 4A depicts the rate of oxidation of dopamine (**1**) to aminochrome (**2**) by H_2_O_2_ in the presence of different concentrations of dopamine, using the different hGQ/DBA nucleoapzyme structures shown in Fig. 3. For the structures displayed in panel I, structures **0**, **4r**, **5r**, and **6r**, (Fig. 3), containing no tether or a flexible strand between the hGQ DNAzyme and the L/L′ duplex unit, the activities were comparable with each other, and revealed only slightly higher activities as compared to the activity of the hGQ strand **ss9** alone. Specifically, *k*_cat_ values for **4r–6r** ranged from (5.9 ± 0.7) × 10^–3^ s^–1^ to (7.2 ± 0.4) × 10^–3^ s^–1^, whereas the single-strand extended hGQ-DNAzyme (**ss9**) corresponds to *k*_cat_ = (4.4 ± 0.2) × 10^–3^ s^–1^ (Table 1, entries 9–12). Importantly, similar to **ss9** the curves obtained with structures **4r**, **5r**, and **6r** do not display typical saturation kinetics (Fig. 4B), which would be expected if a binding site was involved in the catalytic conversion. Thus, for these three systems a *K*_M_ could not be determined, revealing that the binding site was not involved in the oxidation reaction. Only in the case of system **0**, where there is no linker unit between the double-stranded region and the hGQ unit (P_*i*_ = 0), a typical saturation kinetics curve is observed with *k*_cat_ = (7.3 ± 0.4) × 10^–3^ s^–1^ and *K*_M_ = 1.1 ± 0.2 μM (Table 1, entry 1). The low *K*_M_ value showed that system **0** reached *V*_max_ already at low substrate concentrations, indicating that the catalytic reaction reaches saturation with small amounts of dopamine. Clearly, connecting the hGQ and the DBA units with double-stranded DNA in a rigid supramolecular DNA structure allows the interaction between the binding site and catalytic site, although this specific structure does not lead to an optimal positioning of the hGQ DNAzyme with respect to the dopamine binding site. It should be noted that a triplex-stabilized hGQ–DBA nucleoapzyme that includes only one triplex bridge revealed an activity similar to the activity of a nucleoapzyme lacking the triplex bridge. This result shows that a single triplex bridge is too weak to stabilize the active structure of the nucleoapzyme.

**Table 1:** Kinetic parameters of the various supramolecular nucleoapzyme structures with respect to the oxidation of dopamine (**1**) to aminochrome (**2**) in the presence of H_2_O_2_^*a*^

Entry	Code	*k*_cat_ ^*b*^ (10^–3^ s^–1^)	*K*_M_ (μM)	*V*_max_ (μM min^–1^)	*k*_cat_/*k*_2_^*c*^
1	**0**	7.3 ± 0.4	1.1 ± 0.2	0.32 ± 0.02	8.1
2	**3t**	9.4 ± 0.5	1.7 ± 0.3	0.42 ± 0.02	10.4
3	**4t**	27.0 ± 1.0	6.7 ± 0.7	1.20 ± 0.04	30.0
4	**5t**	16.5 ± 0.7	3.7 ± 0.5	0.73 ± 0.03	18.3
5	**6t**	17.0 ± 0.8	5.0 ± 0.7	0.76 ± 0.04	18.9
6	**7t**	12.8 ± 0.3	3.5 ± 0.3	0.57 ± 0.01	14.2
7	**8t**	12.3 ± 0.3	7.0 ± 0.5	0.55 ± 0.01	13.6
8	**9t**	5.2 ± 0.4	4.0 ± 1.0	0.23 ± 0.02	5.8
9	**4r**	6.1 ± 0.5	c.n.b.d.^*d*^	0.27 ± 0.02	6.7
10	**5r**	5.9 ± 0.7	c.n.b.d.^*d*^	0.26 ± 0.03	6.5
11	**6r**	7.2 ± 0.4	c.n.b.d.^*d*^	0.32 ± 0.02	7.9
12	**ss9**	4.4 ± 0.2	c.n.b.d.^*d*^	0.19 ± 0.01	4.9

^*a*^Conditions: 0.5–64 μM dopamine, 100 μM H_2_O_2_; 0.74 μM active catalyst; buffer: 50 mM MES, pH = 5.5, 200 mM KCl, 2 mM MgCl_2_.

^*b*^ *k*_cat_ = *V*_max_/[catalyst] = *V*_max_/0.74.

^*c*^The rate constant for the hGQ DNAzyme (**1**) is: *k*_2_ = (0.9 ± 0.1) × 10^–3^ s^–1^.

^*d*^c.n.b.d. stands for ‘could not be determined’.

Aiming to achieve a higher activity of supramolecular structures in which the catalytic site was positioned in close proximity to the binding site, *i.e.* at the opening of the binding pocket, we studied the hGQ-catalyzed oxidation of dopamine (**1**) to aminochrome (**2**) in the presence of the supramolecular nucleoapzyme structures displayed in panel II (Fig. 3). In order to position the hGQ unit at the opening of the binding pocket, we applied triplex DNA units that would fix the P_*i*_ unit of the hGQ strand to the stem-region of hairpin A. This would position the hGQ DNAzyme closer to the substrate binding pocket. Indeed, when compared to the dopamine oxidation rate of the system in which P_*i*_ was absent (*i* = 0, **0**), the rate was slightly higher for the system with *i* = 3 (**3t**): *k*_cat_ (**0**) = (7.3 ± 0.4) × 10^–3^ s^–1^ and *k*_cat_ (**3t**) = (9.4 ± 0.5) × 10^–3^ s^–1^, respectively (Table 1, entries 1 and 2). The low *K*_M_ values of 1.1 ± 0.2 μM and 1.7 ± 0.3 μM for systems **0** and **3t**, respectively, show that these nucleoapzyme structures become saturated already at low substrate concentrations. Remarkably, increasing the length of the P_*i*_-unit with one triplex-forming nucleotide resulted in a large increase in the activity, *k*_cat_ (**4t**) = (27.0 ± 1.0) × 10^–3^ s^–1^. This value corresponds to a 30-fold increase in the oxidation rate as compared to the hGQ DNAzyme alone, and a 3.7- and 2.9-fold higher activity than systems **0** and **3t**, respectively. Furthermore, comparison of the activity of nucleoapzyme **4t** to the oxidation activity of **ss9** alone or **ss9** and the DBA aptamer unit alone, we find that **4t** reveals only a 5–7-fold catalytic enhancement as compared to the control systems. This is attributed to the fact that the positively charged dopamine substrate reveals non-specific affinity to oligonucleic acids, thus resulting in a local concentration of dopamine in the **ss9** structure that yields its observed catalytic activity. In addition, the high *K*_M_ value of 6.7 ± 0.7 μM reveals that this supramolecular nucleoapzyme structure converts more substrate molecules than systems **0** and **3t** before it is saturated; for this system, *V*_max_ is reached at high substrate concentrations. When compared to the previously described equidimensional **4r** system, the 4.4-fold difference in catalytic activity in favor of the triplex-stabilized system **4t** is remarkable since it is the result of only two nucleotides that were switched in position. In order to ensure that the enhanced activity was not caused by an enhancement of the activity of the hGQ DNAzyme itself, we measured the DNAzyme-catalyzed oxidation of ABTS^2–^ to ABTS˙^–^ by H_2_O_2_ (Fig. S1, ESI†). Since all systems display very similar oxidation rates, we conclude that the differences in the rates of dopamine oxidation did not arise from enhanced activities of the hGQ DNAzyme itself, but rather by the designed proximity of the DBA to the hGQ catalytic site prevailing in **4t** and non-existing in **4r** (*vide infra*). Furthermore, we observed that increasing the length of P_*i*_ with either one or two additional triplex-forming nucleotides resulted in a small drop in the catalytic rate. Specifically, for systems **5t** and **6t**, the observed activities are comparable, *i.e. k*_cat_ (**5t**) = (16.5 ± 0.7) × 10^–3^ s^–1^ and *k*_cat_ (**6t**) = (17.0 ± 0.8) × 10^–3^ s^–1^. Apparently, the hGQ catalytic site and DBA binding site are optimally aligned only in the system with P_*i*_ = 4 (**4t**) nucleotides, and sub-optimally in the systems with P_*i*_ = 5 (**5t**) and P_*i*_ = 6 (**6t**) nucleotides, provided that the P_*i*_-unit is able to form a triplex-DNA structure (Fig. 4B). Also, for these two systems, the curves display saturation kinetics and the relatively high *K*_M_ values of 3.7 ± 0.5 μM for **5t** and 5.0 ± 0.7 μM for **6t** reveal that the binding site is directly involved in the conversion of the substrate to the product. As could be expected, elongating P_*i*_ further resulted in decreasing activities of the nucleoapzyme structures. Specifically, systems that contain P_*i*_ = 7 (**7t**), P_*i*_ = 8 (**8t**), and P_*i*_ = 9 (**9t**), all of which contain the five triplex-forming bases that were already present in **6t** in addition to the extra bases, have lower *k*_cat_ values, *i.e. k*_cat_ (**7t**) = (12.8 ± 0.3) × 10^–3^ s^–1^, *k*_cat_ (**8t**) = (12.3 ± 0.3) × 10^–3^ s^–1^, and *k*_cat_ (**9t**) = (5.2 ± 0.4) × 10^–3^ s^–1^. This drop in the catalytic activity confirms that an optimal alignment of the hGQ DNAzyme and the DBA is achieved by a triplex-forming P_*i*_ = 4 unit (as in **4t**), and that the addition of nucleotides leads to a decrease in activity. In fact, the activity of system **9t** is comparable to that of **ss9**, indicating that in this configuration the DBA-strand (**6**) is not directly involved in the dopamine oxidation process. Although the determined *K*_M_ values seem to indicate that these **7t–9t** systems can convert multiple substrates before *V*_max_ is reached, the lower *V*_max_ and shapes of the curves revealed that the binding site became less involved in the catalytic reaction moving from **7t** to **8t** and **9t**.

### CD spectroscopic evidence of triplex DNA

The presence of the triplex unit was evidenced from the CD spectroscopic analysis and thermal denaturation studies of triplex DNA-containing system **6t** and the non-triplex containing system **6r**. It should be noted that the current triplex-stabilized nucleoapzyme structures contain both Hoogsteen motifs, *i.e.* C–G·C^+^ triplexes that form at acidic pH and T–A·T triplexes that are less pH dependent.^31^ Due to the fact that the dopamine-oxidation studies were performed at pH 5.5, formation of both the C–G·C^+^ and T–A·T triplexes was anticipated. Indeed, in the **6t** system that contains five triplex units, a negative narrow band was observed in the CD spectrum at 213 nm, which is typical for a triplex DNA unit.^32^ Due to the presence of a strong positive band at 210–220 nm caused by the G-quadruplex,^33^ this triplex feature was best visualized when the spectrum of **6t** was subtracted from that of **6r** (Fig. 4C). Further support to the stabilization of the triplex-bridged hemin/G-quadruplex–dopamine binding aptamer structure was obtained by examining the melting curves of the triplex stabilized structure **6t** and of the non-triplex stabilized structure **6r** (ESI, Fig. S2†). The two structures reveal a low-temperature melting process at 43 °C, attributed to the dissociation of the two smallest hairpin structures associated with the DBA unit. The structure **6r** exhibits a second melting curve at 60 °C that corresponds to the separation of the duplex DNA, L/L′. The triplex-stabilized structure **6t** reveals a higher melting process at 64 °C. These results are consistent with the cooperative stabilization of the duplex domain by the triplex binding units. Based on these results, we confirm that the triplex-based alignment of the hGQ unit with respect to the DBA unit is involved in the significantly enhanced activity of the supramolecular structures in the H_2_O_2_-mediated dopamine oxidation reaction.

### Computational simulations of the supramolecular nucleoapzyme systems

**Fig. 5:**
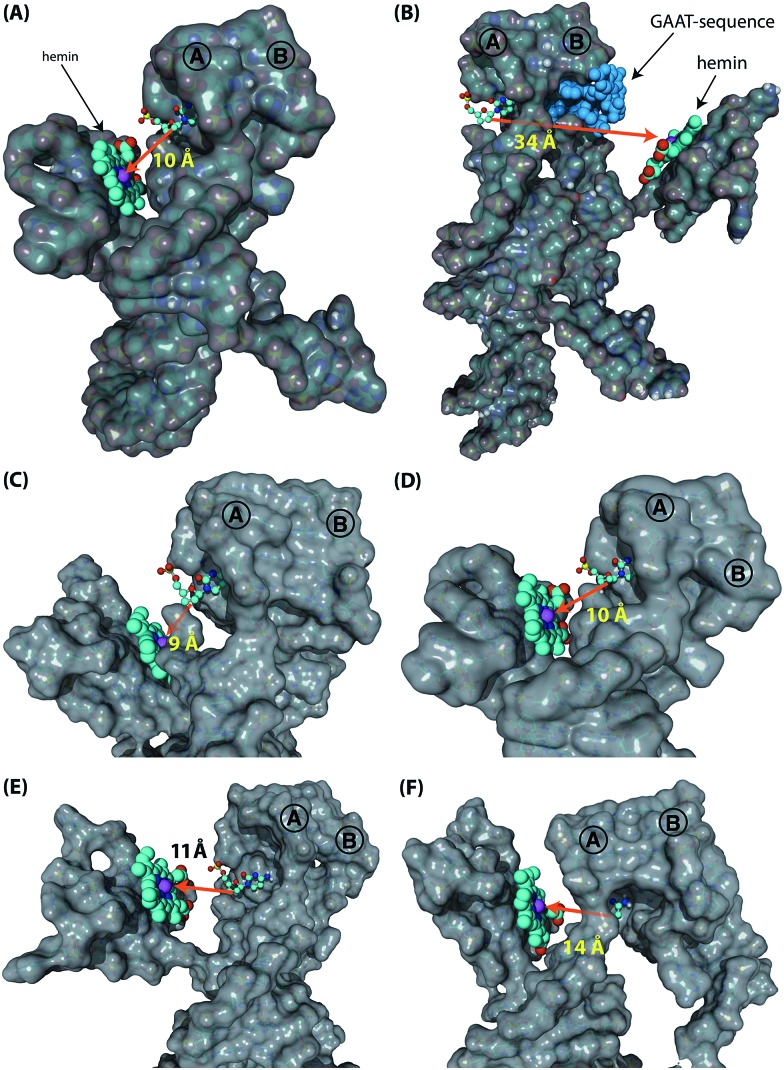
Computational simulations of the various supramolecular nucleoapzyme systems. In all systems, hairpins A and B are indicated, the hemin group is depicted in the space-fill presentation (indicated by the arrows in (A) and (B)), with the catalytically active iron(iii) unit depicted by the purple sphere. The opening of the dopamine binding site is highlighted by the cytosine residue that is depicted in the ball-and-stick presentation (the surface of this residue is removed for clarity). (A) and (B) Models of triplex-stabilized supramolecular system **4t** (A), and of non-stabilized system **4r** (B). In system **4r**, the GAAT-sequence that blocks the 3′-end facing side of the binding pocket, *i.e.* the non-accessible side, is highlighted in blue (the surface of these residues are removed for clarity). (C)–(F) Zoomed versions of the models of **3t** (C), **4t** (D), **5t** (E), and **6t** (F), showing the differences in distance and relative position of the hGQ DNAzyme catalytic unit with respect to the opening of the dopamine binding site.

Following these results on the catalytic performance of the systems, we performed computational simulations of the different systems in order to identify plausible origins of the observed differences in activities.^34^ We were particularly interested to determine the alignment between the hGQ DNAzyme catalytic site and the DBA binding site as a function of (i) the triplex-stabilized units *versus* the non-stabilized units, and (ii) the length of the P_*i*_-unit. First, we studied the most active system **4t** and its substantially less active flexible counterpart **4r** to assess the effect of the triplex-DNA feature on the alignment of the hGQ catalytic unit and the DBA binding pocket. Our computational simulations indicate that the triplex DNA unit formed by the P_*i*_ domain and the stem of hairpin A in structure **4t** stabilizes the interaction between the separate DBA and DNAzyme units. As can be seen in the model, this positions the hGQ DNAzyme active site at the opening of the DBA binding pocket (Fig. 5A). Since the local concentration of dopamine is enhanced at the binding site, positioning the catalytic unit in close proximity of the binding site favors its catalytic conversion. In a previous study, we found that the oxidation product of dopamine, aminochrome, has a poor affinity for the binding site, allowing it to diffuse away from the nucleoapzyme structure and enabling another dopamine substrate to associate with the binding pocket.^28^ In the absence of the triplex DNA unit, the hGQ DNAzyme is not anchored to the stem of hairpin A, and thus it is not positioned at the opening of the binding pocket, but rather occupies a more remote location where it faces the closed side of the binding pocket, *i.e.* on the side of hairpin B (Fig. 5B). Specifically, the distance between the catalytically active iron(iii) center and the C2-atom of the cytosine residue that is positioned at the opening of the binding pocket is increased from 10 Å to 34 Å. Even more, the actual effective distance is even larger as our simulations indicate that the substrate cannot leave the binding pocket *via* hairpin B. Thus, these computational simulations support the *a priori* anticipated preferred alignment of the hGQ active site with respect to the substrate binding site in the triplex-stabilized structure, and present a likely cause for the observed enhanced activity of system **4t** when compared to **4r**. Computational simulations of **5r** and **6r** show that these systems adopt a similar arrangement as shown for **4r** (see Fig. S3, ESI†). Secondly, to rationalize the differences in *k*_cat_ values of the various triplex-stabilized series (**3t–9t**), the series was subjected to computational simulations and the distances between the active site and binding site in the respective structures were measured (Fig. 5C–F). In this analysis, we evaluated not only the distance between opening of the binding pocket and the catalytic unit, but also take into account their relative positioning. Regarding the first, the distance between the two atoms mentioned above is in the range of 9–14 Å for **3t–6t** (Fig. 5C–F); moving to **7t** it increases to 25 Å, for **8t** it is 29 Å, and for **9t** it increased to 33 Å (see Fig. S4, ESI†). Therefore, the decrease in activity when consecutively moving from **6t** to **7t**, **8t**, and **9t** can readily be rationalized by the increased distance between the active site and binding site. However, for the difference in activity between systems **3t–6t**, analysis of the position of the hGQ DNAzyme active site relative to the opening of the binding pocket provides insight for the origin of the different *k*_cat_ values. In system **3t**, the length of P_*i*_ is insufficient to position the active center of the hGQ DNAzyme at the opening of the binding pocket, even though the distance between Fe(iii) and the cytosine residue is slightly shorter than in system **4t**. Rather, it positions the hGQ unit behind the stem of hairpin A (Fig. 5C), explaining the low observed activities of this system. As was mentioned above, in system **4t**, the active center is positioned at the opening of the binding pocket (Fig. 5D). In systems **5t** and **6t**, the hGQ DNAzyme unit is rotated slightly and occupies a more distant less optimal position when compared to **4t** (Fig. 5E and F), explaining the lower activities when compared to system **4t**.

### Switchable catalysis

**Fig. 6:**
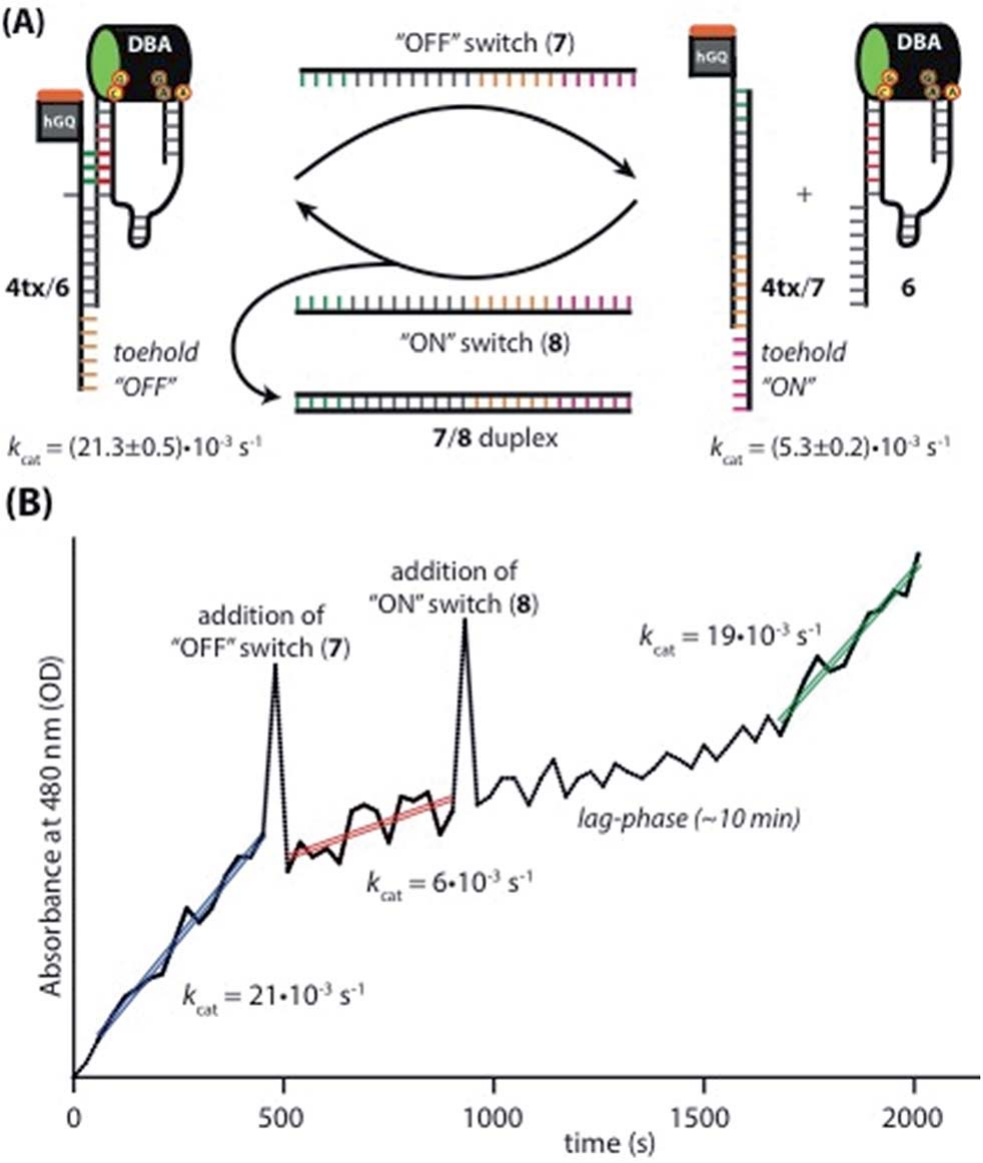
(A) Schematic depiction of the switching supramolecular nucleoapzyme system. hGQ-strand **4tx** contains an “OFF”-toehold that allows the “OFF”-switch (**7**) to separate the catalytic unit and the substrate binding unit. The “OFF”-switch contains an “ON”-toehold that allows the “ON”-switch (**8**) to remove the “OFF”-switch (**7**), which leads to the formation of the catalytic most active species **4tx**/**6**. The different activities of the “ON” and the “OFF” system are given below the structures. (B) Switching the catalytic performance of the system while detecting the absorption of aminochrome (**2**) at 480 nm. The spikes observed in the absorbance values upon the addition of the switching strands originate from momentary perturbation of the reaction mixture originating from the opening of the spectrophotometer lid and the mixing of the added strand solution.

Taking advantage of the programmable nature of DNA and the differences in activity of **4t** when compared to **ss9**, a system with potential switchable catalytic properties was designed. For this, strand L′ was extended with an oligonucleotide tether consisting of eight bases, resulting in strand **4tx** (Fig. 6A). By applying the DBA-containing strand **6**, a supramolecular structure is formed that contains a toehold in order to allow separation of **4tx** from **6** by adding the fuel strand (**7**), a process that should switch “OFF” the activities of the nucleoapzyme. Strand **7** contains also a toehold, which allows the catalytic system to be switched “ON” again by applying the counter strand (**8**) that releases **4tx** from the **7**/**4tx** duplex. Upon removal of strand **7** from **4tx**, the exposed single strand of **4tx** hybridizes again with the full L-unit of the DBA-strand (**6**). This re-activates the catalytic system and leads to higher rates for the oxidation of dopamine (Fig. 6B). Indeed, the oxidation of dopamine in the presence of **4tx**/**6**, *i.e.* the supramolecular hGQ DNAzyme DBA binding site nucleoapzyme structure, reveals a high activity, *k*_cat_ (**4tx**/**6**) = (21.3 ± 0.5) × 10^–3^ s^–1^ while separation of the system to **4tx**/**7** and **6** components yields a substantially lower oxidation rate, *k*_cat_ (**4t**/**7**, **6**) = (5.3 ± 0.2) × 10^–3^ s^–1^ (see Fig. S5 and Table S1, ESI†). Indeed, starting from the system in the “ON” configuration, *k*_cat_ = 21 × 10^–3^ s^–1^, the activity of the system was switched “OFF” upon the addition of strand (**7**), resulting in *k*_cat_ = 6 × 10^–3^ s^–1^. When the counter stand (**8**) was added to the system, its catalytic functions were regenerated. The regeneration of the superior catalytic functions of the system upon addition of (**8**) is associated with a lag-time of 10–15 min. This lag-period is attributed to a slow formation of the triplex-DNA interaction due to the bulky hGQ unit: at room temperature, it takes time to position the hGQ catalytic unit in the for catalysis optimal position. Here we note that the absorption spectrum of the final mixture, as well as HPLC experiments indicated formation of aminochrome (**2**) as the sole product within the time-interval for this experiment. Furthermore, CD spectral analyses support the time-dependent formation of the catalytically switched “ON” triplex-stabilized nucleoapzyme structure. Indeed, after a time-interval of 15 minutes the characteristic triplex band around *λ* = 215 nm was observed (see Fig. S6, ESI†). Lastly, we note that the *K*_M_ values of the **4tx**/**6** and **4tx**/**6** + **7**/**8** differ slightly, while all the other kinetic parameters of the two systems are similar (Table S1†). This is attributed to non-specific affinity of dopamine to DNA. This process reduces the bulk concentration of dopamine, especially at low concentrations, giving rise to the resulting higher *K*_M_ value of the **4tx**/**6** + **7**/**8** system when compared to the **4tx**/**6** system alone (for further discussion, see ESI, Fig. S5 and Table S1†).

## Conclusions

The previously reported approach to design nucleic acids of enhanced catalytic functions, nucleoapzymes, *via* the covalent conjugation of catalytic nucleic acids (DNAzymes) with sequence-specific nucleic acid binding sites (aptamers), has now been extended to the rational design of nucleoapzymes by the duplex and duplex/triplex integration of the DNAzyme/aptamer moieties into functional structures of enhanced catalytic properties. We emphasize, however, that the approaches introduced in the present study are not just an “extension” of the nucleoapzyme concept, but they highlight the versatility provided by nucleic acids to generate new functional DNAzyme/aptamer structures. Specifically, the present study has implemented the hemin/G-quadruplex (hGQ) and the dopamine binding aptamer (DBA) as functional units that catalyse the H_2_O_2_-mediated oxidation of dopamine (**1**) to aminochrome, (**2**). We have demonstrated that by the rational design of duplex-integrated hGQ/DBA units and duplex/triplex-integrated hGQ/DBA units, nucleoapzymes of superior catalytic properties were developed. The significance of spatial steric alignment of the hGQ catalytic site in respect to the DBA binding site, and the rigidification of the nucleoapzyme structure, play important roles in the emerging catalytic properties of the nucleoapzyme. Furthermore, our studies reveal that molecular dynamic simulations provide an effective tool to rationalize the experimental results with computed structures that fit well with the observed catalytic features of the different nucleoapzymes. The significance of the study rests on the following aspects: (i) the systems demonstrate the versatility of using the information encoded in the base sequence of DNA (base pairing, triplex assembly) to construct the catalytic DNAzyme–aptamer conjugates. One may, however, envisage DNA/aptamer nucleoapzymes conjugated by other supramolecular DNA motives, *e.g.* G-quadruplexes, i-motif, Y-shaped or holiday cross-over junctions. Such nucleoapzymes that contain a high level of structural complexities could provide means to mimic protein-based enzymes. (ii) The concepts introduced for the rational design of the hGQ/DBA system could be adapted for the assembly of other DNAzyme/aptamer nucleoapzyme systems. Furthermore, one may implement man-made homogeneous catalysts as catalytic units^35^ coupled to aptamer sites thereby leading to an entirely new paradigm of catalysis in aqueous environments. (iii) Molecular dynamic simulations provided aided tools to rationalize the catalytic features of the nucleoapzymes. This suggests that such simulations could provide, in the future, structural guidelines to assemble nucleoapzymes of improved activities.

## Experimental section

### DNA sequences

The following DNA sequences were used in this study; they were obtained from IDT technologies. The residues that form the dopamine binding site in DBA are shown underlined and bold, and the triplex-forming section in the stem in hairpin A is shown in red bold. Sections in the hGQ-strands that form the third strand in the triplex unit are shown in green bold, while those in the non-triplex forming strands are shown in blue bold.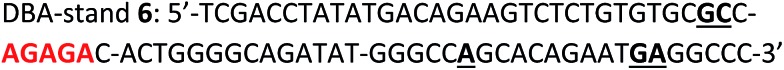 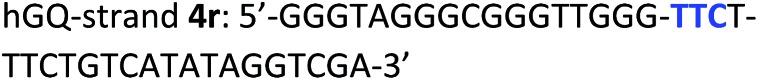 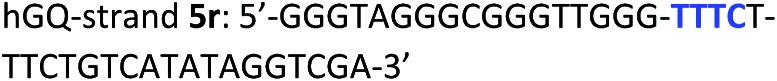 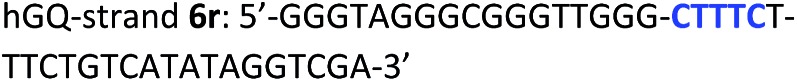 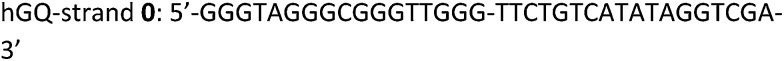 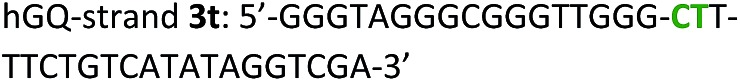 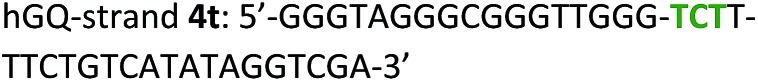 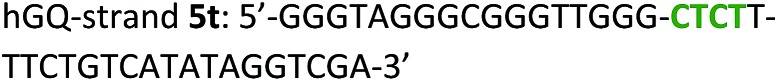 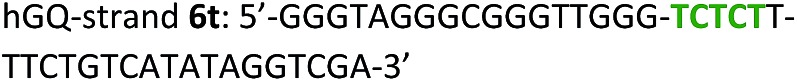 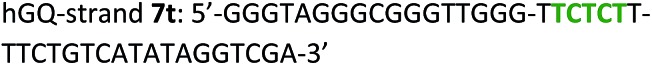 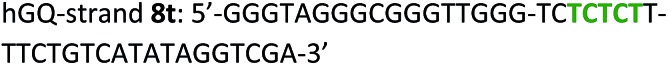 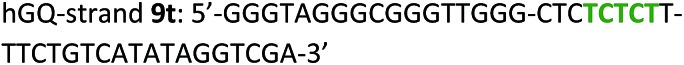 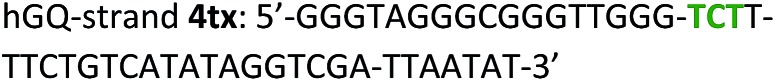 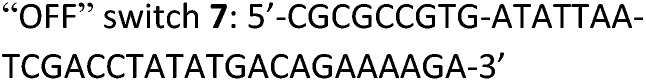 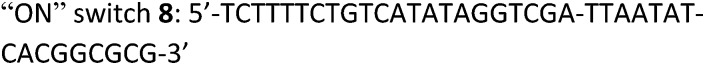


### Oxidation studies

The oxidation of dopamine (**1**) to aminochrome (**2**) in the presence of H_2_O_2_ (100 μM) and the various supramolecular nucleoapzyme structures were performed in MES buffer (5 mM, pH 5.5, 200 mM KCl, 2 mM MgCl_2_) using a plate reader (BioTek Hybrid H1). Duplicate experiments were performed in a sterile half-area microtiter plate (Costar, polystyrene) to ensure the exact same conditions for all systems. Prior to the experiments, a 10 μM solution of the appropriate mixtures of DNA was prepared and annealed at 85 °C for 10 min. Then, the DNA was incubated for 30 minutes at 25 °C after which 1 equiv. of hemin was added; hemin was taken from a freshly prepared stock solution in DMSO (100 μM). Immediately after mixing, 10 μL of the mixture was applied to the wells of a 96-well plate, and 70 μL of the MES buffer was added. This mixture was incubated for 30 minutes at room temperature after which the absorbance spectrum (*λ* = 300–500 nM) of each well was measured in order to determine appropriate formation of the hemin/G-quadruplex DNAzyme. For each of the mixtures, similar Soret bands were observed. Then, 10 μL of various concentrations of dopamine were applied, resulting in the following final concentrations of dopamine: 0.5, 0.75, 1, 1.5, 2, 3, 4, 6, 8, 16, 32, and 64 μM. Lastly, 10 μL of a stock solution of 1 mM H_2_O_2_ was added (final concentration of H_2_O_2_ = 100 μM). The oxidation of dopamine (**1**) to aminochrome (**2**) was monitored at 480 nm, while drifting of the baseline was corrected by measuring the absorbance at 800 nm; time-interval for the data-point collection was set at 2 minutes. The experiments were performed at 25 °C. The reaction rate was determined using the initial straight section of the time-dependent increase in absorbance using the molar extinction coefficient of aminochrome (*ε* = 3058 M^–1^ cm^–1^).^36^

### Switchable catalysis

The oxidation of dopamine (**1**) to aminochrome (**2**) by means of the switchable system was performed as follows. The **4tx**/**6** system was assembled by annealing the separated GQ (**4tx**) and DBA (**6**) units in MES buffer (5 mM, pH 5.5, 200 mM KCl, 2 mM MgCl_2_). Then, 1 equiv. of hemin was added and the system was again incubated for 30 minutes in order to form the catalytic site. After this, 96 μL of the solution containing 1.25 μM of the switchable supramolecular nucleoapzyme structure was transferred to a quartz cuvette, and 12 μL of a 500 μM solution of dopamine and 12 μL of a 1 mM solution of H_2_O_2_ were added. This resulted in the following final concentrations: 1 μM hGQ/DBA, 50 μM dopamine, 100 μM H_2_O_2_. After the oxidation of dopamine (**1**) to aminochrome (**2**) was followed for 8 minutes, 1 equiv. of strand **7** was added. The absorbance of the mixture at 480 nm was measured for 5 minutes before 1 equiv. of the “ON” switch **8** was added. Now, the formation of aminochrome (**2**) was followed for 20 minutes. During the entire experiment, drifting of the baseline was corrected by measuring the absorbance at 800 nm, and subtracting this value from the absorbance measured at 480 nm.

### CD spectroscopy

CD spectra (*λ* = 200–300 nm) were recorded on a Jasco J-810 circular dichroism spectropolarimeter (JASCO Ltd.) at a rate of 500 nm min^–1^ using 200 μL of 1 μM DNA solutions in a 1 cm fused quartz cuvette. Measurements were performed in MES buffer (5 mM, pH 5.5, 200 mM KCl, 2 mM MgCl_2_). To facilitate comparisons, CD spectra were background subtracted, averaged over five measurements, and smoothened.

### Computational simulations

The model of the DBA was constructed as described before.^28^ Using MC-Fold/MC-Sym^37^ and the YASARA Structure software package (version 14.8.17)^38^ the double-strand L/L′ domain was obtained. The hemin/G-quadruplex structure was derived from a published structure of a porphyrin/G-quadruplex system.^39^ Coordinates for the triplex DNA features were extracted from the Protein Data Bank.^40^ The separate units were connected *in silico* in order to obtain the supramolecular nucleoapzyme structures; for the triplex containing systems, atomic coordinates obtained from published triplex-DNA structures were applied in order to construct the P_*i*_-tether (for **3t**, **4t**, **5t**, and **6t**). For the systems that did not contain triplex DNA (**0**, **4r**, **5r**, and **6r**), or the systems that contained a non-hybridized DNA strand on top of the triplex units (**7t**, **8t** and **9t**), the required strands were built using the YASARA Structure software. In order to energy-minimize the constructed systems, a molecular dynamics refinement simulation of the model was run over the time-course of 500 ps using the AMBER03 force field;^41^ for this, the simulation cell was automatically filled with water (density: 0.997 g L^–1^, pH 5.5)^42^ and the content of the cell was neutralized using KCl ions. The structures shown in Fig. 5, S3 and S4 in the ESI† were obtained after this 500 ps long molecular dynamics refinement simulation. Molecular graphics were created with YASARA (; http://www.yasara.org) and POVRay (; http://www.povray.org).

## Supplementary Material

Supplementary information
