# Supplementary material for: Rational design of supramolecular hemin/G-quadruplex–dopamine aptamer nucleoapzyme systems with superior catalytic performance
Source: Chem Sci. 2016 Jan 25;7(5):3092–101. doi: 10.1039/c5sc04832j (PMC6005209; doi:10.1039/c5sc04832j)
Supplement: Supplementary file 1 [file SC-007-C5SC04832J-s001.pdf]

Electronic Supplementary Material (ESI) for Chemical Science.
This journal is © The Royal Society of Chemistry 2016

Supporting Information

for

Rational Design of Supramolecular Hemin/Q-Quadruplex-Dopamine Aptamer Nucleoapzyme Systems with Superior Catalytic Performance

by

H. Bauke Albada, Eyal Golub and Itamar Willner

Table Of Contents page

Oxidation of $ABTS^{2-}$ to $ABTS^{\bullet-}$ by nucleoapzyme structures 3t–9t 2

Melting curves of supramolecular nucleoapzymes 6t and 6r 2

Computational simulations of the 5r and 6r systems 3

Computational simulations of the 7t, 8t, and 9t systems 3

Saturation kinetics curves for the three different states in the switchable system 4

CD spectroscopic analysis of the switching event from 4tx/7 + 6 to 4tx/6 + 7/8 5

Oxidation of $ABTS^{2-}$ to $ABTS^{\bullet-}$ by nucleoapzyme structures **3t–9t**

The oxidation of $ABTS^{2-}$ to its radical anion $ABTS^{\bullet-}$ was performed in a microtiterplate (BioTek Hybrid H1 platereader). Kinetics of the oxidation reactions were followed by monitoring the appearance of the radical anion ($ABTS^{\bullet-}$) at 414 nm over a time-period of 10 min. Reactions were initiated by the addition of H_2O_2 (100 µM) to the $ABTS^{2-}$ (50 µM) solution in the presence of the various nucleoapzyme systems. The measurements were performed in duplicate.

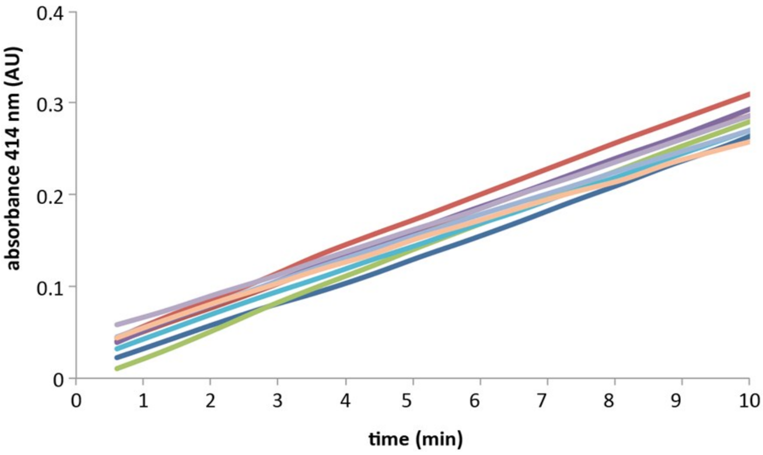

Figure S1. Time-dependent increase in the absorbance of $ABTS^{\bullet-}$ as a result of the supramolecular nucleoapzyme-catalysed H_2O_2-mediated oxidation of $ABTS^{2-}$.

Melting curves of supramolecular nucleoapzymes **6t** and **6r**

Thermal denaturation profiles for **6t** and **6r** were measured using 1 µM DNA in 50 mM MES buffer (pH = 5.5, 200 mM KCl, 2 mM $MgCl_2$). The temperature was raised with 1 °C/min. For both systems, two melting points were observed: $T_{m,1}$ 43 °C (for **6t** and **6r**), and $T_{m,2}$ = 60 °C (for **6r**) and $T_{m,2}$ = 64 °C (for **6t**). The first melting point is assigned to the dissociation of the small hairpins in the DBA unit, the second melting point is assigned to the dissociation of the larger double-strand DNA unit that is applied to merge the GQ unit and the DBA unit L/L'. The presence of the five triplex-forming bases in **6t** stabilize this interaction, when compared to the non-stabilized system **6r**, leading to the higher observed melting point.

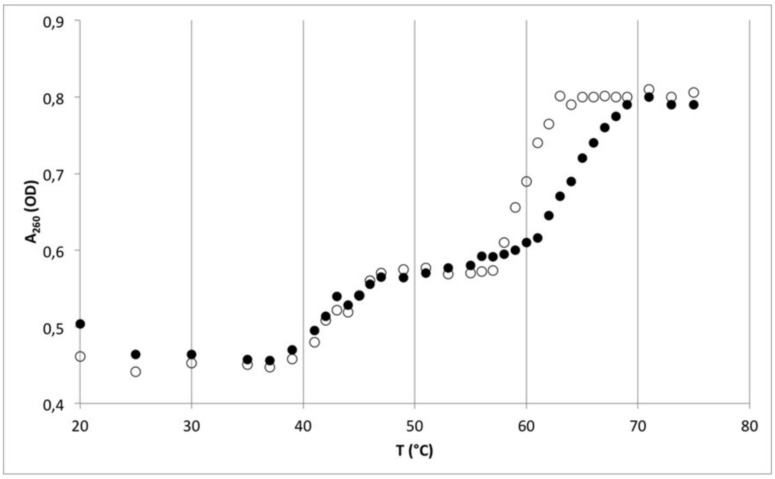

Figure S2. UV melting profile of **6t** (solid circles) and **6r** (open circles) at 260 nm in 50 mM MES, pH = 5.5, 200 mM KCl, 2 mM $MgCl_2$. Concentration DNA = 1 µM.

Computational simulations of the **5r** and **6r** systems

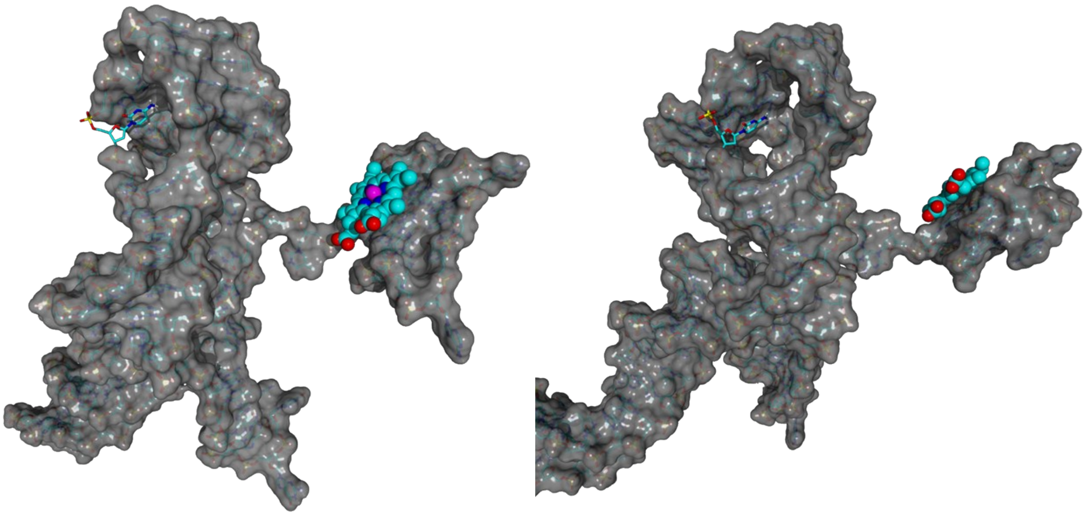

Figure S3. Models of non-stabilized supramolecular system systems **5r** (*left*) and **6r** (*right*).

Computational simulations of the **7t**, **8t**, and **9t** systems

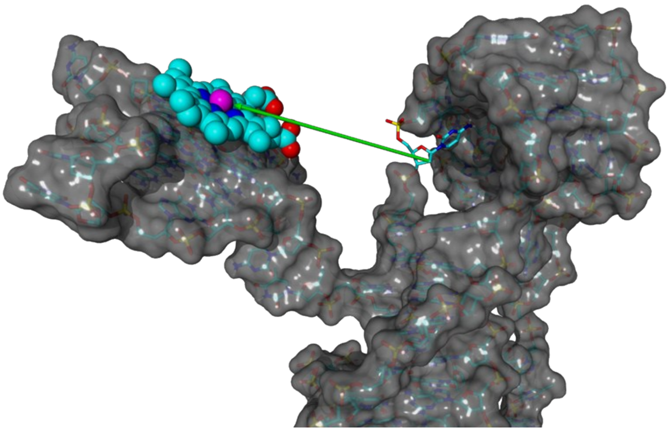

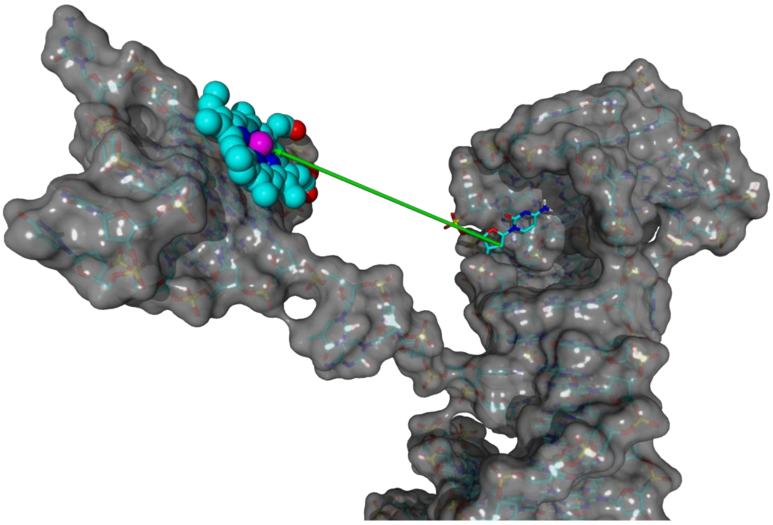

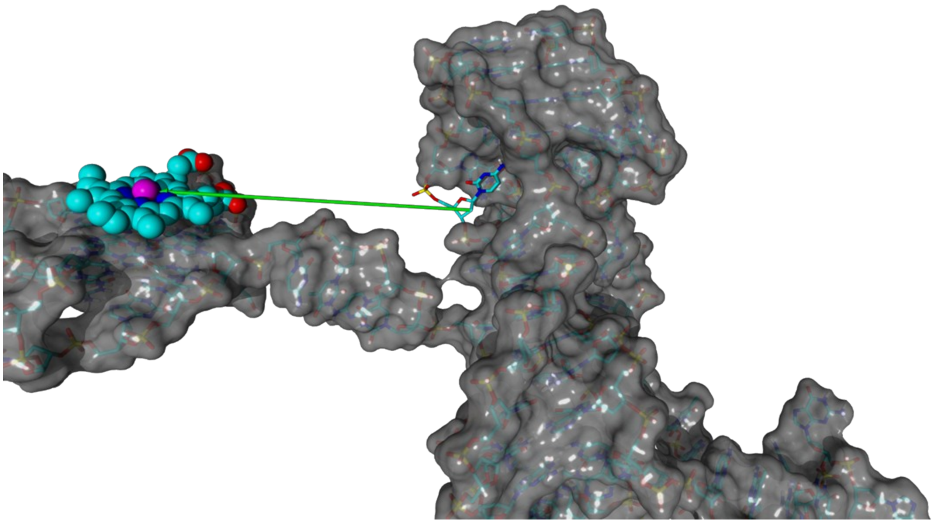

Figure S4. Models of triplex-stabilized supramolecular system systems **7t** (*top*), **8t** (*middle*), and **9t** (*bottom*). Lengths of the arrows are: 25 Å, 29 Å, and 33 Å, for **7t**, **8t**, and **9t**, respectively.

Saturation kinetics curves for the three different states in the switchable system

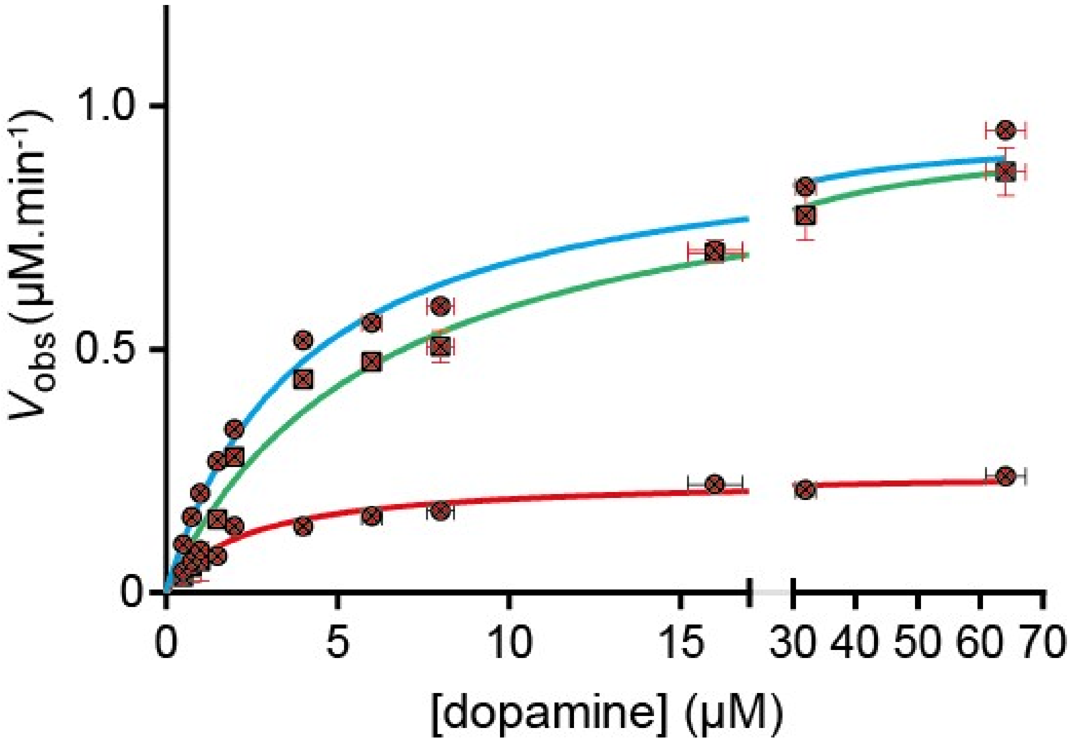

Figure S5. Kinetics curves of the switchable supramolecular nucleoapzyme system **4tx**/**6** (*blue*), separated entities **4tx**/**7** and **6** (*red*), and system **4tx**/**6** in the presence of duplex **7**/**8** (*green*).

Tables S1. Kinetic parameters of the various supramolecular nucleoapzyme structures with respect to the oxidation of dopamine (**1**) to aminochrome (**2**) in the presence of H_2O_2.

System	k_{cat} (10^{-3} s^{-1})[a]	K_M (μM)	V_{max} (μM•min^{-1})	k_{cat}/k_2[b]
4tx/6	21.3 ± 0.5	4.0 ± 0.3	0.95 ± 0.02	23.7
4tx/7	5.3 ± 0.2	2.3 ± 0.3	0.24 ± 0.01	5.9
4tx/6 + 7/8	21.2 ± 0.7	6.2 ± 0.7	0.95 ± 0.03	23.6

Conditions: 0.5–64 μM dopamine, 100 μM H_2O_2; 0.74 μM active catalyst; buffer: 50 mM MES, pH = 5.5, 200 mM KCl, 2 mM $MgCl_2$. *Notes*: [a] $k_{cat} = V_{max}$ / [catalyst] = V_{max} / 0.74. [b] the rate constant for the hGQ DNAzyme (**1**) is: $k_2 = (0.9 \pm 0.1)\cdot 10^{-3}$ s^{-1}.

We note that for the system **4tx/6** and **4tx/6** + **7/8** the K_M values differ by ca. 50% while all other kinetic parameters are similar (k_{cat}, V_{max}). This apparent difference in the K_M values is attributed tot the fact that dopamine reveals non-specific affinity towards duplex DNA (in this case dsDNA **7/8**). Since K_M values are predominantly determined by the rates (v) at relatively low concentrations of dopamine, this non-specific binding of dopamine in the **4tx/6** + **7/8** mixture leads to a lower substrate concentration for the nucleoapzyme at these lower concentrations, and thus to a higher K_M value. This fact is, also, reflected by the lower V_{obs} values for the **4tx/6** + **7/8** system when compared to the **4tx/6** system alone, specifically at the lower dopamine concentration regiment (at ≤8 μM dopamine). At high concentrations of dopamine (at ≥16 μM dopamine), at which the V_{max}-values are calculated, this side-effect binding is negligible, leading to similar V_{max} values of the two systems.

CD spectroscopic analysis of the switching event from **4tx/7** + **6** to **4tx/6** + **7/8**

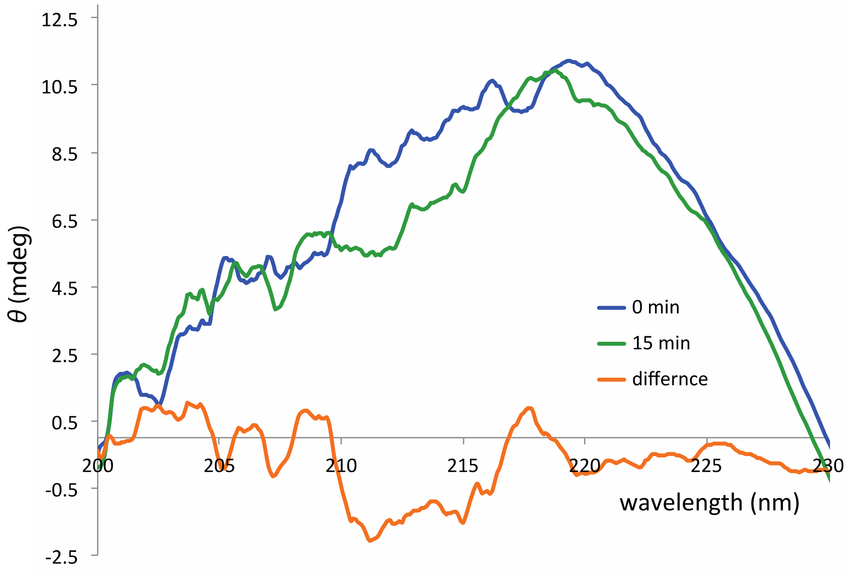

Figure S6. CD spectra of **4tx/6** + **7/8** obtained 0 and 15 min after addition of "ON" switch (**8**) to the mixture of **4tx/7** + **6**. Appearance of the negative feature around 213 nm shows the time-dependent formation of the triplex feature.
